# Temperature related infectious disease mortality among infants and seasonality in Sweden, 1868-1892

**DOI:** 10.1093/eurpub/ckac131.154

**Published:** 2022-10-25

**Authors:** J Junkka, M Hiltunen

**Affiliations:** Centre for Demographic and Ageing Research, Umeå University, Umeå, Sweden

## Abstract

**Background:**

Climate conditions, such as ambient temperature, are crucial to infants’ vulnerability to infectious diseases. However, little is known about how climate conditions, such as temperature and seasonality, affects infectious disease mortality among infants in high mortality settings. The aim was to investigate the association between ambient temperature, seasonality and cause-specific infant mortality.

**Methods:**

We applied a retrospective study design using parish register data from Sweden covering the period 1868-1892 in combination with daily temperature data. Population data and temperature data were combined in a time-series dataset, accounting the number of deaths per day by age group. Mortality due to water- and foodborn diseases, airborne infectious diseases, and other causes were modelled as a function of temperature exposure in the previous 14 days using distributed lagged non-linear models.

**Results:**

We found that airborne infectious disease mortality was not related to cold temperatures but rather to seasonality. At the 1st of february IIR was 2.98 (CI 1.30 - 6.85). The summer mortality peak due to water- and foodborne infections were associated with high temperatures and not with seasonality. At + 20 °C (the 99th percentile temperature exposure) IRR was 5.52 (CI 3.13-9.74).

**Conclusions:**

The increased vulnerability to infectious diseases of infants at high temperatures is a significant future risk, given the expected global warming in the coming decades.

**Key messages:**

• Airborne infectious disease mortality was related to seasonality while water- and foodborne infectious diseases were related to high temperatures.

• The increased vulnerability to infectious diseases of infants at high temperatures is a significant future risk, given the expected global warming in the coming decades.

